# Why do I hear but not understand? Stochastic undersampling as a model of degraded neural encoding of speech

**DOI:** 10.3389/fnins.2014.00348

**Published:** 2014-10-30

**Authors:** Enrique A. Lopez-Poveda

**Affiliations:** ^1^Audición Computacional y Psicoacústica, Instituto de Neurociencias de Castilla y León, Universidad de SalamancaSalamanca, Spain; ^2^Grupo de Audiología, Instituto de Investigación Biomédica de Salamanca, Universidad de SalamancaSalamanca, Spain; ^3^Departamento de Cirugía, Facultad de Medicina, Universidad de SalamancaSalamanca, Spain

**Keywords:** auditory deafferentation, aging, hearing loss, speech intelligibility, stochastic sampling, auditory encoding, hearing impairment, speech processing

## Abstract

Hearing impairment is a serious disease with increasing prevalence. It is defined based on increased audiometric thresholds but increased thresholds are only partly responsible for the greater difficulty understanding speech in noisy environments experienced by some older listeners or by hearing-impaired listeners. Identifying the additional factors and mechanisms that impair intelligibility is fundamental to understanding hearing impairment but these factors remain uncertain. Traditionally, these additional factors have been sought in the way the speech spectrum is encoded in the pattern of impaired mechanical cochlear responses. Recent studies, however, are steering the focus toward impaired encoding of the speech waveform in the auditory nerve. In our recent work, we gave evidence that a significant factor might be the loss of afferent auditory nerve fibers, a pathology that comes with aging or noise overexposure. Our approach was based on a signal-processing analogy whereby the auditory nerve may be regarded as a stochastic sampler of the sound waveform and deafferentation may be described in terms of waveform undersampling. We showed that stochastic undersampling simultaneously degrades the encoding of soft and rapid waveform features, and that this degrades speech intelligibility in noise more than in quiet without significant increases in audiometric thresholds. Here, we review our recent work in a broader context and argue that the stochastic undersampling analogy may be extended to study the perceptual consequences of various different hearing pathologies and their treatment.

## Introduction

Hearing impairment is a serious and growing disease: its prevalence worldwide is around 11% for adults; around 280 million people have hearing impairment; and adult-onset hearing impairment is the third leading cause of disability (Stevens et al., 2013).

Typically, hearing impairment is said to occur when audiometric thresholds averaged over frequencies 0.5, 1, 2, and 4 kHz are at least 35 decibels (dB) higher than normal (Stevens et al., 2013). Hearing impairment is thus defined using a detectability (or audibility) criterion rather than a speech intelligibility criterion. This is paradoxical considering that hearing impaired listeners rate their difficulty at understanding speech, particularly in noisy environments, as the most limiting aspect of their hearing impairment (Kochkin, 2002). Also paradoxical is that hearing impairment is defined based on the **audiogram**, that is, on a rough measure of detectability of sound energy across frequencies, even though speech is a dynamic, time-varying stimulus and much of its information is conveyed in the changes of its energy over time (Diehl et al., 2004).

KEY CONCEPT 1. Audiogram A graph showing the detection threshold intensity for pure tones as a function of tone frequency. Typically, intensity is expressed as hearing loss in decibels.

Obviously, audibility affects intelligibility: when speech cannot be heard, it cannot be understood. The ability to understand speech in quiet environments may be reasonably predicted with the **speech intelligibility index**, a measure of the spectral components of speech that are above the listener's audiometric thresholds (ANSI S3.5, 2007). Less obvious is, however, that reduced intelligibility is *not only* and *not always* associated with reduced spectral detectability (Moore, 2007). Evidence of this is that the speech intelligibility index is sometimes insufficient to accurately predict intelligibility, particularly in the presence of sound “jammers” (Woods et al., 2013). Further evidence is that hearing aids restore audibility and yet hearing aid users still rate speech-in-noise intelligibility as the number-one improvement sought in hearing aids, over speech-in-quiet intelligibility (Kochkin, 2002). Further evidence is that elderly listeners with clinically normal audiograms show less than normal speech-in-noise intelligibility (CHABA, 1988; Peters et al., 1998; Pichora-Fuller and MacDonald, 2008). In other words, the intelligibility of speech in noisy environments must depend upon more aspects than just audibility.

KEY CONCEPT 2. Speech intelligibility index A measure of the speech spectrum that is audible. Each spectral region is given a weighting according to its contribution to intelligibility.

Traditionally, these additional aspects have been sought in the way the speech spectrum is encoded in the pattern of impaired mechanical cochlear responses. Given the dichotomy between **Spectral and temporal cues** in speech perception, more recent studies, are steering the focus toward how the speech waveform is encoded in the auditory nerve. A potentially crucial factor is the loss of primary auditory nerve fibers, or deafferentation (Kujawa and Liberman, 2009). The focus of this review is a study where we reasoned that deafferentation combined with the stochastic nature of auditory nerve discharges can degrade speech-in-noise intelligibility without affecting audiometric thresholds (Lopez-Poveda and Barrios, 2013). Our approach was based on a signal-processing analogy whereby the auditory nerve may be regarded as a stochastic sampler of the sound waveform and deafferentation may be described in terms of waveform undersampling. This analogy offers an interesting conceptual framework within which to study the perceptual consequences of various different hearing pathologies and their treatments.

KEY CONCEPT 3. Spectral vs. temporal speech cues A sound may be regarded as a distribution of energy over frequency (a spectrum) or as a distribution of energy over time (a waveform). It is controversial whether intelligibility is based on the audible portions of the speech spectrum, on the audible features of speech waveform, or on a combination of both.

## Mechanisms of speech encoding in the cochlea

The cochlea, a snail shaped structure in the inner ear, functions like an auditory prism separating the frequency components of the incoming sound so that they stimulate different populations of auditory neurons. Each region along the length of the cochlea may be described as acting as an acoustic filter tuned to a particular sound frequency and with a certain bandwidth. The cochlea as whole may be described as acting as a bank of such filters functioning in parallel.

The characteristics of cochlear filters strongly depend upon the physiological status of outer hair cells (OHCs), a specialized type of cells in the inner ear. OHCs amplify mechanical cochlear responses to low-intensity sounds. This **Cochlear amplifier** contributes to our exquisite auditory sensitivity. Prolonged exposure to intense sounds or treatment with some ototoxic drugs can damage OHCs or even reduce their number. OHC loss or dysfunction reduces mechanical cochlear sensitivity to low-intensity sounds (Ruggero et al., 1990). This causes an audiometric loss accompanied by important side effects that might degrade the encoding of supra-threshold speech in noisy environments.

KEY CONCEPT 4. Cochlear amplifier A mechanism within the cochlea that provides acute sensitivity in the mammalian auditory system. Key to the normal functioning of this mechanism is the physiological status of outer hair cells, a specialized type of cells in the organ of Corti.

A first side effect of OHC dysfunction is broadened cochlear filters (Robles and Ruggero, 2001). Cochlear filters are more sharply tuned in the healthy cochlea than in a cochlea with OHC damage. Broadened cochlear filters can smear the cochlear representation of the acoustic spectrum, making it harder to separately perceive the frequency components of the target speech from those of interfering sounds, hence hindering speech-in-noise intelligibility (Baer and Moore, 1993).

A second side effect of OHC dysfunction is reduced suppression. In the healthy cochlea, the cochlear response to a sound may be suppressed (reduced) by simultaneous sounds with neighboring frequencies. Suppression might facilitate speech-in-noise intelligibility by enhancing the most salient frequency features of the target speech against those of the background noise (Deng and Geisler, 1987; Young, 2008). OHC dysfunction reduces suppression and this might hinder speech-in-noise intelligibility.

A third side effect of OHC dysfunction is reduced compression. In the healthy ear, cochlear filters apply greater gain at low than at high acoustic intensities, and thereby compress a wide range of acoustic intensities into a narrower range of mechanical responses (Robles and Ruggero, 2001). This compression accounts for the wide dynamic range of human hearing (Oxenham and Bacon, 2003) and might also facilitate the understanding of speech in interrupted or fluctuating noise by amplifying the speech in the silent noise intervals, a phenomenon known as “dip listening.” OHC dysfunction linearizes cochlear responses and reduces compression, which might hinder “dip listening” (Gregan et al., 2013).

A last side effect of OHC dysfunction is reduced efferent control of cochlear function. OHCs receive efferent input from neurons in the medial olivary complex of the auditory brain. When activated, these efferents reduce cochlear mechanical sensitivity and thus cause a mild loss of audibility but they could improve speech-in-noise intelligibility by increasing the discriminability of transient sounds in noisy backgrounds (Kim et al., 2006b; Brown et al., 2010; Guinan, 2010).

Given the demonstrated fragility of OHCs and that OHC dysfunction causes an audiometric loss accompanied by the above described side effects, the explanation for the reduced speech-in-noise intelligibility of hearing impaired listeners has traditionally focused mainly on faulty cochlear mechanics. While seemingly reasonable, this thinking is almost certainly only partially correct. First, for hearing impaired listeners, there is no significant correlation between residual cochlear compression and the benefit from “dip listening” (Gregan et al., 2013), which undermines the role of compression on the intelligibility of supra-threshold speech in noisy backgrounds. Second, at high intensities, cochlear tuning is comparable for healthy and impaired cochleae (Robles and Ruggero, 2001) and yet hearing impaired listeners still perform more poorly than do normal-hearing listeners on speech-in-noise intelligibility tests (reviewed in pp. 205–208 of Moore, 2007). Third, age *per se* degrades speech-in-noise intelligibility, even for listeners with clinically normal audiometric thresholds and presumably healthy OHCs (CHABA, 1988; Peters et al., 1998; Kim et al., 2006a). Fourth, the reduced speech intelligibility of hearing impaired listeners appears to be associated with their inability to use the information conveyed in the rapid temporal changes of speech sounds, known as “temporal fine structure” (Lorenzi et al., 2006; Bernstein et al., 2013).

Altogether, this evidence suggests that the poorer-than-normal speech-in-noise intelligibility of hearing impaired listeners is not only (or not mostly) due to impaired cochlear mechanics or to a degraded representation of the speech spectrum in the pattern of mechanical cochlear responses. Instead, the evidence points to other physiological mechanisms that probably reduce the listeners' ability to encode and/or process the rapid temporal changes in speech. The idea that intelligibility relies on a waveform code is not new. It was favored by early studies that showed that the representation of the speech spectrum in terms of the discharge rate of populations of auditory nerve fibers degenerates at high intensities while a representation based on the temporal aspects of the discharge pattern is stable across sound intensities (Young and Sachs, 1979; Young, 2008) (further evidence is given by Loebach and Wickesberg, 2008, and Shannon et al., 1995). Recent studies, however, have revealed some mechanisms that can degrade the neural representation of the speech waveform, and whose detrimental effects on intelligibility are more significant in noise than in quiet.

## The nerve's perspective

The human auditory nerve contains around 30,000 fibers (Makary et al., 2011) each of which is tuned in frequency roughly following the tuning of the cochlear region it innervates (Narayan et al., 1998). Animal studies show that auditory nerve discharges generally occur in synchrony with the peaks of the sound waveform (Rose et al., 1971). Although the strength of the synchronization decreases with increasing sound frequency, some synchronization still occurs for frequencies up to about 12 kHz (Recio-Spinoso et al., 2005).

Henry and Heinz (2012) have shown that in chinchillas, acoustic trauma reduces the amount of synchronization to a pure tone only when the tone is embedded in noise but not when it is presented in quiet. Acoustic trauma causes a hearing loss associated with broader auditory nerve frequency tuning presumably by damage to the OHCs. Henry and Heinz argued that in noise, the more broadly tuned fibers in the impaired ear “capture” comparatively more noise than tone signal. Hence, the temporal pattern of auditory nerve discharges conveys comparatively more information about the noise than about the tone in the impaired than in the healthy ear. If human speech intelligibility were based on the encoding of speech in the temporal pattern of auditory nerve discharges, as seems to be the case (Stevens and Wickesberg, 1999; Loebach and Wickesberg, 2008), this mechanism might explain the poorer-than-normal speech-in-noise intelligibility of hearing impaired listeners.

This mechanism is particularly interesting because it somewhat reconciles the (more traditional) cochlear- and spectral-centered theory of hearing impairment with the (more recent) evidence that hearing impaired listeners suffer from diminished access to speech temporal cues. However, it has been questioned that broader cochlear tuning *per se* contributes to impaired speech-in-noise intelligibility (Ching and Dillon, 2013). Furthermore, the mechanism demonstrated by Henry and Heinz (2012) still does not explain why elderly listeners with normal audiometry, and presumably normal cochlear tuning, still show poorer-than-normal speech-in-noise intelligibility.

Pichora-Fuller et al. (2007) argued that aging probably reduces the temporal synchrony of neural discharges at different levels of the auditory system and showed that in humans, temporally “jittering” the frequency components in speech degrades speech-in-noise intelligibility with negligible degradations in audibility or long-term spectral cues. This suggests that older listeners with clinically normal audiometry may suffer from impaired speech-in-noise intelligibility due to reduced temporal synchrony of auditory nerve discharges.

In the following sections, we review our proposed mechanism that could contribute to reduced speech-in-noise intelligibility both for audiometrically normal, aged listeners and for hearing-impaired listeners (Lopez-Poveda and Barrios, 2013).

## The auditory nerve as a stochastic sampler of the sound waveform

Pooling speech-evoked spike trains from many auditory nerve fibers appears to be effective for encoding sounds in both frequency and time (Stevens and Wickesberg, 1999). A stimulus waveform is reasonably well-represented in the population nerve response over a wide range of levels (Delgutte et al., 1998) and frequencies (Heinz et al., 2001). On the other hand, individual auditory nerve discharges are stochastic events that may occur (or not) depending on certain probability rules (e.g., Sumner et al., 2002). Roughly speaking, a discharge is more likely to occur for intense than for soft sounds (e.g., Figure 1 in Heil et al., 2011). Inspired by this, we proposed that each auditory nerve fiber operates as a **binary, stochastic sampler** of the sound waveform^1^, and that the quality of the representation of a sound's waveform in the population auditory nerve fiber response would depend both on the probability of firing of individual fibers *and* on the number of available fibers. Therefore, a reduction in either the probability of firing of individual fibers or in the number of auditory nerve fibers would degrade the quality of the neural representation of the sound waveform. Using a signal processing analogy, the defects associated with reducing the number of fibers or their individual probability of firing would be akin to **undersampling**. Therefore, we referred to our proposed mechanism as “stochastic undersampling.”

KEY CONCEPT 5. Binary sampler A sampler that outputs a value “1” when an event occurs and a value “0” otherwise. For example, it outputs a value “1” at sampling instances when sound pressure exceeds a particular criterion value and “0” otherwise.

KEY CONCEPT 6. Undersampling A technique where one samples a continuous sound too slowly to accurately represent its high frequency components. Therefore, we referred to our proposed mechanism as “stochastic undersampling.”

**Figure 1:**
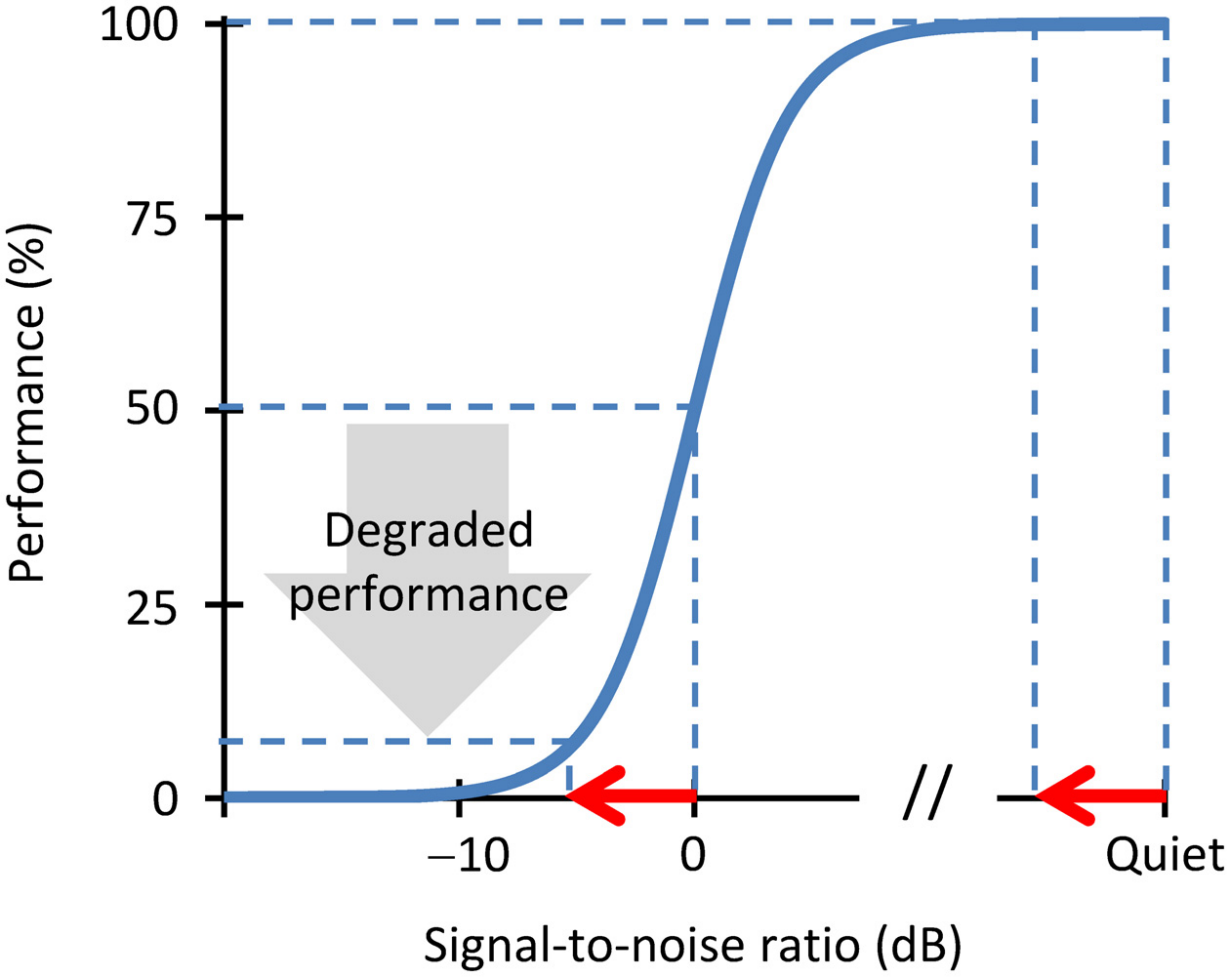
**A schematic illustration of the effects of stochastic undersampling on speech intelligibility in noise and in quiet**. Consider a speech intelligibility task (e.g., the identification of sentences) in different amounts of background noise. The blue trace depicts a hypothetical psychometric function showing performance (the percentage of correctly identified sentences) as a function of the amount of noise, with the latter expressed as the speech-to-noise (SNR) ratio in dB. The speech reception threshold (SRT) is, by definition, the SNR at which the listener correctly identifies 50% of the sentences. Consider now that stochastic undersampling reduces the effective SNR by a fixed amount, depicted by the red arrow. For a speech-in-quiet condition, such an SNR reduction barely degrades performance. By contrast, for a more challenging condition of speech in noise, the same SNR reduction degrades performance significantly.

A crucial aspect of our theory is that neural stochastic undersampling would impair speech intelligibility in noise more than in quiet. This is because the defects of **stochastic sampling** may be described as noise (Dippé and Wold, 1985). Stochastic undersampling would thus yield a noisy representation of the speech signal. Of course, undersampling would also yield a noisy representation of the noise, but noise of noise is noise nonetheless. Therefore, the net effect of undersampling would be a noisier representation of the speech in the auditory nerve; i.e., a reduction of the effective speech-to-noise ratio. This reduction may be sufficient to significantly degrade intelligibility in noise without a significant degradation of intelligibility in quiet. Figure 1 gives a hypothesized explanation of how this might happen.

KEY CONCEPT 7. Stochastic sampling A procedure to extract discrete pressure values (or samples) from a continuous sound at random time intervals. This is in contrast to the standard form of sampling where pressure samples are extracted at regular time intervals.

## Neural stochastic undersampling caused by deafferentation

The ear is a complex organ. Alteration to any of its structures or processes can reduce the probability of firing of individual nerve fibers and hence degrade the encoding of speech by stochastic undersampling. Stochastic undersampling may also occur by **deafferentation**, a reduction in the number of available nerve fibers. The latter is what we explored in the study at the focus of this review (Lopez-Poveda and Barrios, 2013).

KEY CONCEPT 8. Deafferentation A reduction in the number of auditory nerve fibers that send information from the ear to the auditory brain, or of the synapses that those fibers make with cochlear hair cells. In the present context, deafferentation refers particularly to a reduction in the number of afferents in contact with inner hair cells, a specialized type of cells in the ear that transduce sound into nerve discharges.

**Figure 2:**
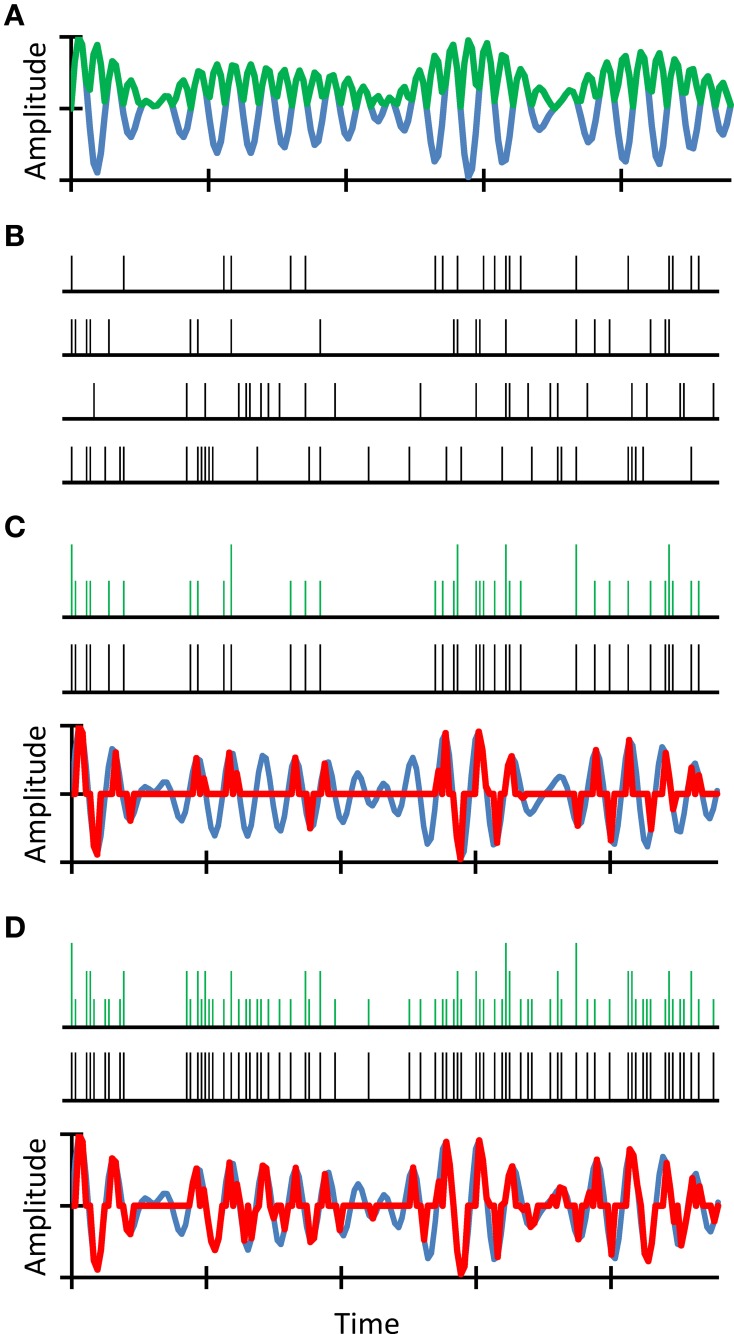
**An example simulation of stochastic undersampling by deafferentation and its consequences on the waveform representation in quiet**. Consider a sound waveform (blue traces in **A,C,D**) and its full-wave rectified (FWR) version (green trace in **A**). Consider also four auditory nerve fibers each of which can fire along the sound waveform following a simple principle: the probability of firing is proportional to the instantaneous sound pressure in the FWR waveform. Since spikes are stochastic events, spike trains are different for the four fibers **(B)**. The green traces in **(C,D)** illustrate neural representations of the sound waveform that result from time-wise summation of only the upper two **(C)** or all four **(D)** spike trains, respectively. Clearly, the sound waveform is better represented in **(D)** than in **(C)**. To illustrate this more clearly, acoustical-waveform equivalents of the aggregated spikes trains are shown as red traces in **(C**,**D)**. These were obtained by time-wise multiplication of the original waveform with an aggregated spike train obtained using a time-wise logical OR function (black spike trains in **C**,**D**). Clearly, the waveform reconstructed using four fibers resembles more closely the original waveform than that reconstructed using only two fibers (compare the red and blue traces in **C**,**D**). In other words, a reduction in the number of fibers degrades the neural representation of the sound waveform. For further details, see (Lopez-Poveda and Barrios, 2013).

We reasoned that intense sounds are more likely to elicit a discharge in an auditory nerve fiber than are soft sounds. Also, because discharges occur stochastically in time, a prolonged, sustained sound is more likely to evoke a discharge than a brief, transient sound of identical intensity. We further reasoned that despite the lower probability of firing of individual fibers to soft or transient sounds, these features still have a good chance to be encoded in the *population* auditory nerve response because the nerve contains thousands of fibers (Stevens and Wickesberg, 1999, 2002). However, a reduction in the number of fibers would reduce the chance that these features be represented in the neural population response. Our rationale is illustrated in Figure 2. Note that our proposed principle is a development of the “volley” theory of hearing (Wever, 1949).

Of course, the probability of firing of individual auditory nerve fibers is actually governed by complex rules that include cochlear amplification, refractoriness of auditory nerve discharges, or reduced synchronization of discharges at high frequencies (Sumner et al., 2002). Furthermore, not all auditory nerve fibers have identical discharge probability functions (Winter et al., 1990; Heil et al., 2011). The point we were making, though, is that the stochastic nature of auditory nerve discharges combined with the number of available fibers imposes a limit to information encoding in the nerve.

**Figure 3:**
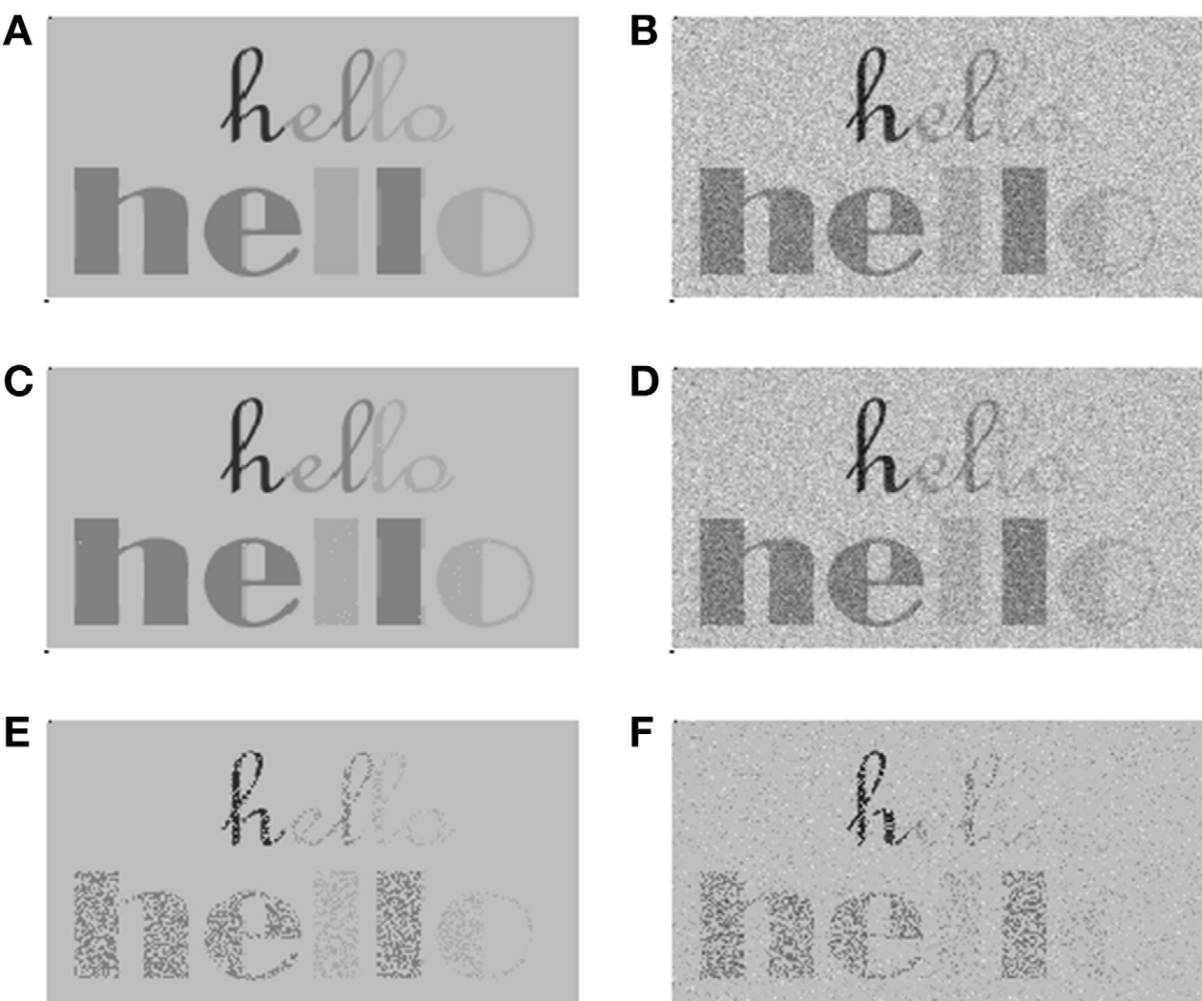
**A visual example to illustrate the consequences of stochastic undersampling of a signal in quiet and in noise**. We used the stochastic sampling principles illustrated in Figure 1 (Lopez-Poveda and Barrios, 2013), whereby the probability of firing is proportional to intensity, or pixel darkness in this example. **(A,B)** The signal in quiet and in noise, respectively. The signal deliberately contains darker and lighter features that would correspond to intense and soft features in speech, respectively. It also contains thick and thin features that would correspond to low- and high-frequency features in speech, respectively. **(C,D)** Stochastically sampled images using 10 samplers per pixel. This number of samplers is sufficient to make this signal intelligible both in quiet **(C)** and in noise (**D**). **(E,F)** Stochastically sampled images using one stochastic sampler per pixel. Now the signal is still detectable and intelligible in quiet **(E)** but less so in noise **(F)**. Particularly degraded are the low-intensity (lighter gray) and high-frequency (thinner lines) features of the signal, like the “lo” portion of the upper “hello” word.

We showed that, as with any other form of stochastic undersampling, the effects of undersampling caused by deafferentation would reduce the intelligibility of speech in noise without a significant reduction of detectability or intelligibility in quiet (Lopez-Poveda and Barrios, 2013). We also showed that the fewer the number of fibers, the greater the amount of sampling noise, the more degraded the neural representation of the speech waveform and the poorer the intelligibility of speech in noise. Figure 3 illustrates this using an intuitive visual example.

We also conjectured that stochastic undersampling caused by deafferentation could explain the poorer-than-normal speech-in-noise intelligibility of elderly and hearing-impaired listeners. It has been recently shown that in human, the number of afferent auditory nerve fibers decreases with increasing age even for listeners with otherwise seemingly normal cochleae (Makary et al., 2011). Therefore, stochastic undersampling by deafferentation could explain the poorer-than-normal speech-in-noise intelligibility of elderly listeners with normal hearing. It has also been shown that after suffering a temporary hearing loss by exposure to intense sounds, mice show a permanent reduction of auditory nerve synapses (Kujawa and Liberman, 2009). Therefore, listeners who develop a permanent hearing impairment by noise exposure are likely to suffer from severe deafferentation. Stochastic undersampling by deafferentation could be the reason why some of these listeners have poorer speech-in-noise intelligibility than normal-hearing listeners or than predicted by the speech intelligibility index.

## Discussion

The reviewed evidence suggests that reduced audibility is only partly responsible for impaired speech intelligibility in noisy environments and that once reduced audibility is accounted for, hearing-impaired listeners still suffer from poorer speech intelligibility in noisy environments compared to normal-hearing listeners. The reason is still uncertain. Recent evidence steers the focus from degraded representations of the speech spectrum in the pattern of impaired cochlear mechanical responses to degraded representations of the speech waveform in the auditory nerve. Two mechanisms may contribute to the latter: reduced synchronization of *individual* auditory nerve fiber discharges (Pichora-Fuller et al., 2007; Henry and Heinz, 2012), and stochastic undersampling (Lopez-Poveda and Barrios, 2013). Reduced synchronization of individual fibers may be associated with aging (Pichora-Fuller et al., 2007) and with audiometric loss (Henry and Heinz, 2012). In either case, its impact is greater in noise than in quiet. Stochastic undersampling can occur by multiple hearing pathologies, including deafferentation, a hearing pathology that comes with aging (Makary et al., 2011) and may or may not be associated with an audiometric loss (Kujawa and Liberman, 2009). As reviewed, the impact of undersampling by deafferentation is also greater in noise that in quiet.

### An alternative conceptual framework to investigate hearing deficits and treatment outcomes

That the auditory nerve operates as a stochastic sampler of the sound waveform is a convenient signal-processing analog of auditory nerve function. Thus far, we have used this analogy to model the perceptual consequences of deafferentation in terms of stochastic undersampling, but stochastic undersampling may also be caused by various different hearing pathologies. Insofar as any hearing pathology may alter the probability of firing of individual auditory nerve fibers, it may also cause stochastic undersampling. For example, OHC dysfunction (without deafferentation) would reduce auditory sensitivity to soft sounds and hence the probability of firing of individual nerve fibers to these sounds. Since the defects of stochastic undersampling may be described as noise, stochastic undersampling by OHC dysfunction would decrease the effective speech-to-noise ratio in the auditory nerve to soft speech features without greatly affecting the representation of intense speech features. Other hearing pathologies not reviewed here may also reduce the probability of firing of individual fibers (or subpopulations of fibers) to different waveform features. In the impaired ear, multiple pathologies can occur simultaneously. A long-standing goal of hearing research is to disentangle the relative importance of different pathologies for speech-in-noise intelligibility. To this end, the stochastic undersampling model offers an alternative conceptual framework in terms of how individual pathologies alter the normal probability of firing of individual nerve fibers and how the “abnormal” firing probabilities degrade the encoding of the stimulus waveform in the population nerve response.

This framework may be applied to explore further perceptual effects of deafferentation. For example, because deafferentation comes with aging (Makary et al., 2011) and because deafferentation degrades the representation of transient waveform features (Lopez-Poveda and Barrios, 2013), the model predicts that older (deafferented) listeners should have more problems at detecting rapid, transient waveform features than younger listeners. This prediction is yet to be tested with our model but it is broadly consistent with the evidence that elderly listeners have difficulty at detecting rapid sound features, as if their hearing was slower than normal (Schneider and Hamstra, 1999).

The model might also be applied to understand across-listener variability of hearing-aid and cochlear-implant outcome. For example, cochlear implants allow the use of fast stimulation rates to convey rapid waveform features to the implant user. According to our model, stochastic undersampling by deafferentation would effectively represent rapid waveform features as noise. This suggests that the use of high rates of electrical stimulation might be beneficial to cochlear implant users with good neural survival and less so (or even detrimental) to users suffering from deafferentation. In other words, deafferentation might be the reason why some listeners prefer lower stimulation rates than others.

### Conflict of interest statement

The author declares that the research was conducted in the absence of any commercial or financial relationships that could be construed as a potential conflict of interest.

## Footnotes

^1^Actually, each fiber would sample the waveform of the mechanical cochlear response for the cochlear region innervated by the fiber.
